# Mental Health Promotion and Intervention in Occupational Settings: Protocol for a Pilot Study of the MENTUPP Intervention

**DOI:** 10.3390/ijerph19020947

**Published:** 2022-01-15

**Authors:** Ella Arensman, Cliodhna O’Connor, Caleb Leduc, Eve Griffin, Grace Cully, Doireann Ní Dhálaigh, Carolyn Holland, Chantal Van Audenhove, Evelien Coppens, Fotini Tsantila, Victoria Ross, Birgit Aust, Arlinda Cerga Pashoja, Johanna Cresswell-Smith, Laura Cox, Lars de Winter, Naim Fanaj, Birgit A. Greiner, Ulrich Hegerl, Sharna Mathieu, Ana Moreno-Alcázar, Wendy Orchard, Charlotte Paterson, György Purebl, Gentiana Qirjako, Hanna Reich, Paul Corcoran

**Affiliations:** 1 School of Public Health, University College Cork, T12 CY82 Cork, Ireland; caleb.leduc@ucc.ie (C.L.); evegriffin@ucc.ie (E.G.); grace.cully@ucc.ie (G.C.); B.Greiner@ucc.ie (B.A.G.); pcorcoran@ucc.ie (P.C.); 2 National Suicide Research Foundation, University College Cork, T12 XF62 Cork, Ireland; cliodhna.oconnor@ucc.ie (C.O.); dnidhalaigh@ucc.ie (D.N.D.); carolyn.holland@ucc.ie (C.H.); 3 Australian Institute for Suicide Research and Prevention, School of Applied Psychology, Griffith University, Brisbane, QLD 4122, Australia; victoria.ross@griffith.edu.au (V.R.); s.mathieu@griffith.edu.au (S.M.); 4 LUCAS, Centre for Care Research and Consultancy, KU Leuven, 3000 Leuven, Belgium; chantal.vanaudenhove@kuleuven.be (C.V.A.); evelien.coppens@kuleuven.be (E.C.); fotini.tsantila@kuleuven.be (F.T.); 5 Academic Center for General Practice, KU Leuven, 3000 Leuven, Belgium; 6 National Research Centre for the Working Environment, 2100 Copenhagen, Denmark; BMA@nfa.dk; 7 Department of Population Health, Faculty of Epidemiology and Population Health, London School of Hygiene and Tropical Medicine, London WC1E 7HT, UK; Arlinda.CergaPashoja@phe.gov.uk; 8 The Equality Unit, Mental Health Team, Finnish Institute for Health and Welfare (THL), FI-00271 Helsinki, Finland; johanna.cresswell-smith@thl.fi; 9 MATES in Construction, Level 1, 35 Astor Terrace, Spring Hill, QLD 4004, Australia; lcox@mates.org.au; 10 Phrenos Center of Expertise, 2016 Utrecht, The Netherlands; lwinter@kcphrenos.nl; 11 Mental Health Center Prizren, College of Medical Sciences Rezonanca, 1000 Prishtina, Kosovo; naimfanaj@gmail.com; 12 European Alliance against Depression e.V., 04109 Leipzig, Germany; ulrich.hegerl@deutsche-depressionshilfe.de; 13 Johann Christian Senckenberg Distinguished Professorship, Department of Psychiatry, Psychosomatic Medicine and Psychotherapy, University Hospital, Goethe University, 60528 Frankfurt am Main, Germany; 14 Centre Fòrum Research Unit, Institut de Neuropsiquiatria i Addiccions, Parc de Salut Mar, 08019 Barcelona, Spain; amoreno.centreforum@gmail.com; 15 Hospital del Mar Medical Research Institute (IMIM), 08003 Barcelona, Spain; 16 International Association for Suicide Prevention (IASP), Washington, DC 20015, USA; wendyorchard@iasp.info; 17 Nursing, Midwifery and Allied Health Professionals Research Unit (NMAHP-RU), University of Stirling, Stirling FK9 4LA, UK; charlotte.paterson@stir.ac.uk; 18 Institute of Behavioural Sciences, Semmelweis University, 1085 Budapest, Hungary; purebl.gyorgy@med.semmelweis-univ.hu; 19 Department of Public Health, Faculty of Medicine, University of Medicine, AL1005 Tirana, Albania; gentaqirjako@gmail.com; 20 German Depression Foundation, 04109 Leipzig, Germany; hanna.reich_de_paredes@deutsche-depressionshilfe.de; 21 Depression Research Centre of the German Depression Foundation, Department of Psychiatry, Psychosomatic Medicine and Psychotherapy, University Hospital, Goethe University, 60528 Frankfurt am Main, Germany

**Keywords:** workplace-based health interventions, organisational interventions, workplace health promotion, process evaluation, mental health and well-being, occupational, depression, suicide, suicidal behaviour, self-harm

## Abstract

Depression and anxiety are the most prevalent mental health difficulties in the EU, causing immense suffering and costing the global economy EUR 1 trillion each year in lost productivity. Employees in construction, health and information and communications technology have an elevated risk of mental health difficulties. Most mental health interventions for the workplace have been targeted at larger companies and small and medium-sized enterprises (SMEs) are often overlooked despite most people being employed in SMEs. The MENTUPP intervention aims to improve mental health and wellbeing and reduce depression, anxiety, and suicidal behaviour. The MENTUPP project involves the development, implementation, and evaluation of a multilevel intervention targeting both clinical and non-clinical mental health issues and combating the stigma of mental (ill-)health, with a specific focus on SMEs. The intervention is underpinned by a framework of how to create a mentally healthy workplace by employing an integrated approach and has been informed by several systematic reviews designed to understand organisational mental health interventions and a consultation survey with key experts in the area. The intervention is facilitated through the MENTUPP Hub, an online platform that presents interactive psychoeducational materials, toolkits, and links to additional resources in an accessible and user-friendly manner. This paper presents the pilot study protocol for delivering the MENTUPP intervention in eight European countries and Australia. Each intervention country will aim to recruit at least 23 participants in 1–3 SMEs in one of the three high-risk sectors. The central aim of the pilot study will be to examine the feasibility, acceptability, and uptake of the MENTUPP intervention across the target SMEs. The findings will contribute to devising the protocol for a cluster randomised controlled trial (cRCT) of the MENTUPP intervention. Findings from this study will also be used to inform the optimisation phase of the MENTUPP intervention which will aim to improve the materials and the implementation of the intervention as well as enhancing the evaluation strategy which will be employed for the cRCT.

## 1. Introduction

A negative working environment may lead to occupational stress and subsequently, physical and mental health problems (including harmful use of substances or alcohol) [1,2]. Research indicates that occupational stress and mental health problems have been linked to absenteeism (the number of days unable to work), presenteeism (reduced ability to work productively and work performance), low employee satisfaction, and lost productivity [1,3,4,5]. Psychosocial stresses in the workplace, such as job uncertainty, emotional demands, poor social relationships, low job control, poor management, harassment and bullying, poor communication, and long working hours, have been shown to undermine mental health [2,6,7,8,9]. Depression and anxiety are the most prevalent mental disorders in the EU [10], costing the global economy EUR 1 trillion each year in lost productivity [11].

There is a strong association between depression and suicidal behaviour (suicide and self-harm), which is compounded by comorbidities, including anxiety and stress-related mental and physical health symptoms [12]. Additionally, it is well-known that depression often co-occurs with burnout and that they can develop simultaneously [13]. A systematic meta-review has indicated that there may be an association between a negative work environment and the development of work-related stress, depression, and anxiety [14]. Another recent meta-review of 72 literature reviews also showed a strong association between psychosocial factors with mental disorders, especially with regard to job strain, long working hours, and effort-reward imbalance [15].

In a systematic review of predictors for burnout, job demands, lack of job resources, lack of social support, social hindrance, poor organisational commitment, high work-family conflict, and poor communication may have harmful effects on occupational burnout [16]. Research has consistently shown that people in specific occupational sectors—construction and health—have mental health problems and an elevated risk of suicide, jointly representing up to 49% of male suicides and 26% of female suicides [17]. For example, research has indicated that doctors and nurses are more likely to experience burnout than other occupations because working overtime is commonplace in the profession [18] and employees who work excessive hours have poorer health overall compared to their counterparts [19]. This is further compounded by the specific emotional demands of the occupation [20]. Furthermore, employees in the construction sector are at increased risk of suicide [21,22,23] with male construction workers in the UK almost four times more likely to die by suicide than the general population [22,24]. Suicide in this sector has been associated with increased alcohol consumption, relationship issues, and poor help-seeking behaviours [25,26]. The construction sector is associated with specific workplace factors, such as high work demands and lack of job control [8,27], which is evident across the organisational structure with supervisors also experiencing high psychological stress [9]. In addition, employees working in the information and communications technology (ICT) sector are at increased risk of depression, comorbid stress related symptoms, and reduced wellbeing in comparison to other occupational sectors because of the high workload, ambitious work targets, and shift-work, associated with the sector [28,29]. Male-dominated workplaces, such as, e.g., the construction and ICT sectors, are also known to have high levels of stigma related to mental health and cultures which encourage self-reliance and stoicism, which may hinder help-seeking behaviour, thus increasing distress [30].

The healthcare, construction, and ICT sectors are associated with high levels of work-related stress, which has been linked to severe, negative psychological outcomes [15,31]. This has been further compounded by the mental health impacts of COVID-19 [32]. The pandemic has been associated with certain mental health issues such as anxiety, post-traumatic stress disorder and sleep disorders in healthcare workers, while factors such as job insecurity and long periods of isolation which are common in the construction and ICT sectors may worsen psychological distress [32].

Within the construction, health, and ICT sectors, employees and managers/owners of small to medium-sized enterprises (SMEs) may be at heightened risk of mental health problems. Many SMEs may have limited capacity to implement mental health promotion or occupational stress programmes [33] because they lack the expertise, knowledge, and budget to implement health promoting programmes [34]. Moreover, they can face specific challenges which employees and managers/owners in larger enterprises may not face including social isolation, long work hours, reluctance to introduce new and safer practices and technologies, unhealthier work environment, lack of human resource management systems, and financial pressures [35,36]. Given that 94% of all enterprises in the European Union are classified as small to medium-sized, SMEs present an ideal opportunity to implement a mental health intervention that promotes mental wellbeing, prevents the development of mental health problems, and tackles stigma associated to mental health.

Workplaces can be a source of well-being [4] and individual employee mental health is inextricably linked to the organisational psychosocial workplace environment [37]. Supportive workplace environments have been linked to lower levels of job dissatisfaction, burnout, and depression [38,39]. Programmes that educate and encourage peer support have been shown to be successful in encouraging help-seeking outcomes [40,41]. Workplaces that promote mental health awareness, de-stigmatise mental illness, and support people with mental disorders are more likely to reduce levels of depression, absenteeism, presenteeism, and increase productivity, and are more likely to benefit from associated economic gains [42,43,44]. Research has shown that improving access to evidence-based interventions for minor stress-related depressive symptoms in occupational sectors associated with high suicide rates may prevent the development of severe depressive disorders and comorbidities, and subsequent suicidal behaviour, and facilitate recovery from mental ill-health [42,45]. ‘Mates in Construction’, a large-scale intervention in the construction sector in Australia, has demonstrated acceptability and effectiveness of a mental health promotion intervention in increasing suicide awareness, sign-posting to relevant support, and help-seeking behaviours [26]. Despite these facts, only 7% of mental health promotion and prevention programmes globally are workplace-based [46].

Workplace interventions directed at both the organisational level and individual level have been shown to result in positive outcomes [45,47], by preventing mental ill-health and by improving job performance [48]. Research indicates that the implementation of workplace mental health interventions may be successful in SMEs [49]. Workplace interventions that adopt an organisational approach, whereby action occurs by management, may have significant, positive effects on mitigating burnout [50,51,52,53,54], decreasing anxiety [55], and alleviating feelings of stress [56,57]. Research shows that management needs to be involved in fitting the intervention into the existing organisation goals, policies, and infrastructure [58]. This can be facilitated through the implementation of a local ‘champion’ to ensure that the intervention fits into the organisation, and importantly, to encourage engagement with the intervention [59]. Action from management targets the psychosocial workplace factors that directly impact on stress, burnout, depressive symptoms, and anxiety symptoms [60]. Mental health promotion should not only be implemented top down via management, but also bottom up, targeting individuals within the organisation [61].

The literature indicates that workplace interventions should be tailored to the resources and needs of the workplace to increase efficacy [62]. This includes employees in planning and implementation to improve the ownership and acceptability of the intervention [63]. Complex workplace interventions require continuous observation, reflection, and adaptation to the implementation strategy in order to be successful [64], therefore a rigid standardised implementation process is not feasible or recommended. While a standardised process may be helpful from a research perspective, its practicality is limited for future generalisability to other settings. At present, there is no known workplace-based intervention that targets mental ill-health on the spectrum of non-clinical to clinical symptoms, and that focuses on each level of the organisation to maximise effectiveness.

Workplace mental health promotion interventions applicable to individuals, groups, and whole populations can be delivered through media and communication technologies [65] and individuals who engage with a mental health intervention regularly can experience improvements in relation to feelings of work stress, and well-being, with lasting effects [66]. Unguided, web-based interventions have also been shown to be effective in improving symptoms of mild/subthreshold depression [67]. The use of digital interventions also has the potential for increased reach, particularly in the context of sectors that have inflexible shift patterns and busy workloads (i.e., healthcare), and that have employees working on various sites (i.e., construction) [68].

The EU Horizon 2020 project Mental Health Promotion and Intervention in Occupational Settings (MENTUPP) is a timely initiative. MENTUPP is a comprehensive, multilevel intervention targeting both clinical mental disorders (depression and anxiety disorders), non-clinical mental health problems (stress, burnout, depressive symptoms), and stigma related to mental health in the workplace. The multilevel intervention adopts the framework to create more mentally healthy workplaces by Petrie et al. [69] and is targeted at the individual level (e.g., providing coping strategies, psychoeducation), group level (e.g., peer-support and de-stigmatisation), the supervisor level (e.g., encouraging help-seeking, addressing psychosocial risks in the work environment), and the organisational level (e.g., promoting positive work environments) [69]. The framework encompasses primary prevention for healthy workers, secondary prevention for symptomatic or at-risk workers, and tertiary prevention for workers with mental illness. The MENTUPP intervention applies an integrated approach to workplace mental health through (i) protecting mental health by reducing work-related and other risk factors for mental health problems, (ii) promoting mental health by developing the positive aspects of work as well as worker strengths and positive capacities, and (iii) responding to mental health problems as they manifest at work regardless of cause (work-related or otherwise) [69]. It addresses leaders and HR staff as gatekeepers, all employees and leaders that are suffering from depression, anxiety or reduced wellbeing or are at-risk for mental ill-health, and the local culture at the SME to promote a healthy working environment and to combat stigma against depression and mental ill-health. The development of an integrated intervention approach such as MENTUPP is likely to achieve maximum mental health benefits, given that organisational changes may result in sustainable transformations in the workplace in addition to addressing mental health issues at an individual level [70] and that employee mental health cannot be viewed in isolation from the workplace psychosocial environment [71]. Becoming active on all levels of intervention with sufficient intensity can stimulate synergistic and catalytic effects in multilevel interventions [72].

Given that interventions are hypothesized to be more successful when tailored to the characteristics of those engaging with them [73], the materials developed for the MENTUPP intervention are tailored to the construction, healthcare, and ICT sectors with a focus on the work-related factors and problems experienced. Intervention materials will be available to employees and managers/owners of SMEs through an online platform known as the MENTUPP Hub where users can self-navigate through the tailored interventions. The materials consist of psychoeducational material delivered via online written materials and videos as well as reflective and practical exercises. Research indicates that psychoeducational interventions can reduce symptoms of depression, anxiety, and psychological distress by informing individuals what actions they can take to prevent, intervene, and treat their symptoms [74], and may offer a first-step intervention to individuals experiencing psychological distress or depression [75]. A variety of methods will be employed to deliver the materials, such as online written materials, videos, audio clips, and reflective and practical exercises, to enhance the usability and acceptability of the Hub for all users [76]. The primary aim of the MENTUPP intervention is to improve mental health and mental wellbeing in the workplace, with a secondary aim of reducing depression and suicidal behaviour by providing tailor-designed mental health promotion materials for the construction, healthcare, and ICT sectors.

The central aim of the pilot study will be to examine the feasibility, acceptability, and uptake of the MENTUPP intervention across target SMEs. This paper describes the protocol for conducting the pilot study. The findings will allow for optimisation of the MENTUPP intervention, implementation, and evaluation in function of the cluster randomised controlled trial (cRCT).

The specific objectives of the pilot study will be to:

○ Evaluate the delivery of the interventions via the MENTUPP Hub;
○ Evaluate the procedures and instruments that will be used to evaluate the MENTUPP intervention;
○ Examine the implementation strategy of the MENTUPP intervention;
○ Estimate parameters required in the power calculations for the MENTUPP cRCT.

## 2. Materials and Methods

### 2.1. Study Design

**Figure 1:**
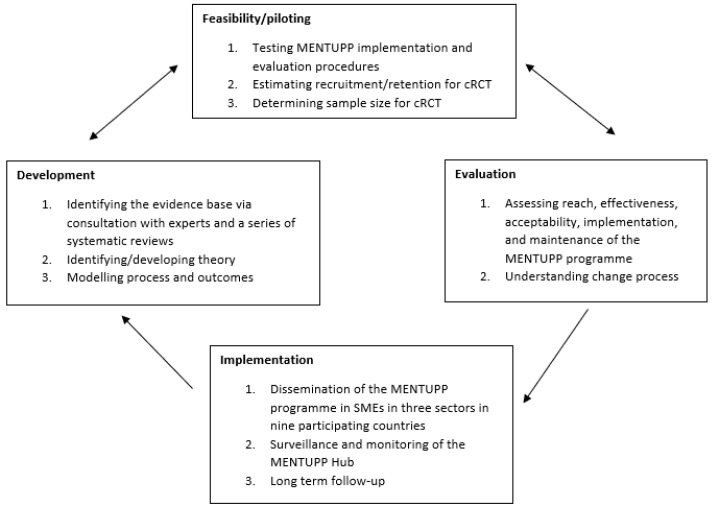
Key elements of the development and evaluation of MENTUPP.

This pilot study will involve both quantitative and qualitative measures to evaluate the delivery of the MENTUPP intervention and relevant tailored materials in the construction, healthcare, and ICT sectors, and within each country using colloquial and everyday language of the intervention country. The study design will be a non-randomised, uncontrolled pre- and post-intervention process evaluation (and preliminary outcome evaluation). MENTUPP is a complex, multilevel intervention [77,78,79] which entails multiple sources of data collection (surveys, a monitoring instrument, log data of users, and focus groups with leaders, employees, and local research officers responsible for implementing the MENTUPP intervention in the intervention countries) to examine the delivery and implementation of the intervention in different organisational and cultural settings. This study represents one aspect (feasibility and piloting) of the broader MENTUPP project which aims to develop, implement, and evaluate a workplace-based, mental health promotion intervention (see Figure 1).

### 2.2. Study Population

#### 2.2.1. Organisation Level

Nine countries will participate in the pilot study and each country will recruit at least one SME from a specific sector. This will be as follows: construction—Albania, Australia, and Ireland; health—Hungary, Kosovo, and The Netherlands; ICT—Finland, Germany, and Spain. Local research officers in each country will recruit one or more SMEs for their specified sector and this will be guided by what is most practical (e.g., approaching an SME with which they already have links, connecting with representatives by establishing a connection with workers’ groups, etc.). A small sized enterprise is defined as an enterprise with between 10 and 50 employees and a medium sized enterprise consists of 50–250 employees [80]. Definitions and inclusion criteria of the construction, health, and ICT sectors are based on NACE guidelines [81]. Construction SMEs include companies involved in site preparation, building of complete structures or parts thereof, civil engineering works, as well as those involved in sub-contracting, materials supply, professional design, engineering, etc. For the pilot study, the focus on healthcare SMES will be on residential care facilities. ICT SMEs are those involved in computer programming, consultancy and related activities, information service activities, and telecommunications.

SMEs will be deemed eligible for the intervention if they agree to predetermined criteria: (1) allocating a local champion to the project, (2) creating a project planning group within the SME with at least one employee and one member of management, (3) allowing employees at least eight hours over six months to use the MENTUPP Hub, and (4) actively and regularly promotion the intervention with employees.

#### 2.2.2. Individual Level

Participants will be deemed eligible for the intervention if they are: (1) full- or part-time employees or either contractors, managers/supervisors, or both, including individuals on sick leave or other types of authorised leave (e.g., maternity leave, care leave); (2) working within an SME in the construction/healthcare/ICT sector; (3) are aged 18 years or older; and (4) are willing to give their informed consent to the study.

#### 2.2.3. Sample Size

One of the aims of this pilot study is to estimate the level of attrition expected in the cRCT [79]. Each of the nine intervention countries will recruit 1–3 SMEs in one of the three sectors (construction/healthcare/ICT) aiming to recruit approximately 60–70 participants (employees and managers) in each intervention country. This sample size allows for an attrition rate of 62–67%, ensuring at least 23 complete sets of data across the intervention (pre- and post-intervention) within each country. With a sample of approximately 600 participants and accounting for 60% attrition, the 95% confidence interval for those lost to post-intervention would be 56–64%, i.e., a margin of error of 4%.

### 2.3. Description of the Study Intervention

The intervention is underpinned by a framework of how to create a mentally healthy workplace [69] by employing an integrated approach [82]. The development of the intervention has been informed by several systematic reviews designed to understand organisational mental health interventions in the construction, healthcare, and ICT sectors, a systematic review to identify the facilitators and barriers to the implementation of mental health promotion interventions delivered in workplace settings [83], and a consultation survey with key stakeholders in the three sectors and academia.

**Figure 2:**
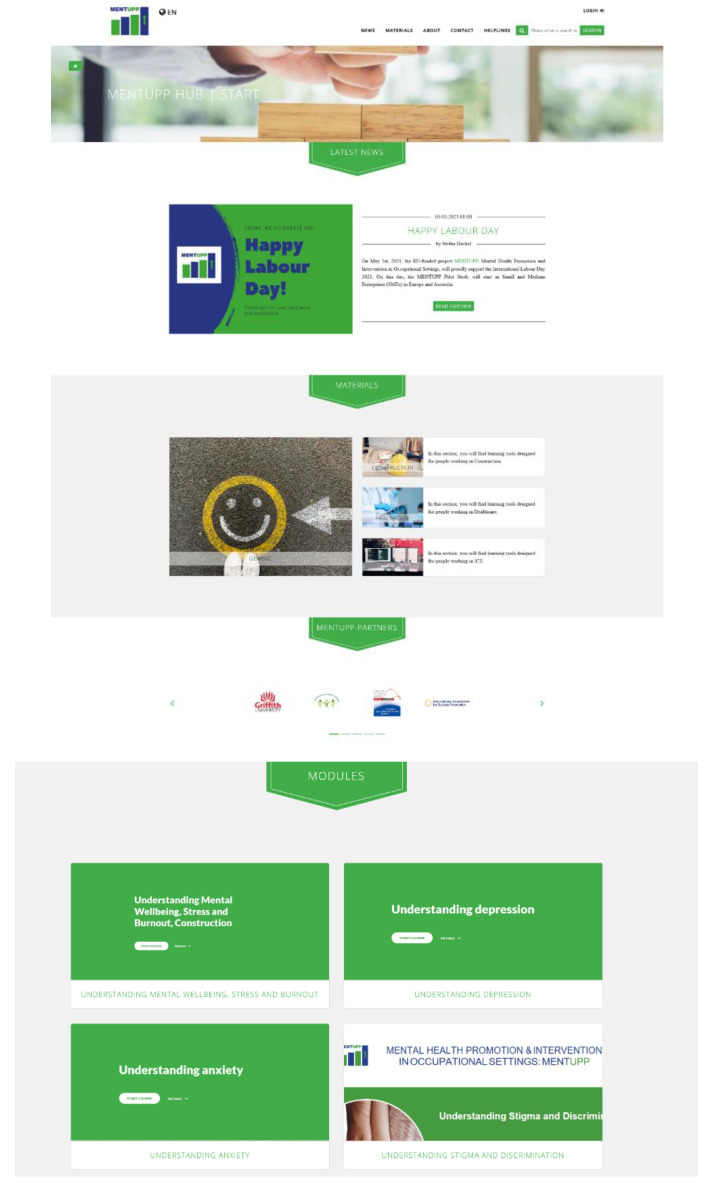
Screenshots of MENTUPP Hub.

The study intervention is designed to improve mental health in the workplace, reduce stigma and promote mental wellbeing. The intervention is facilitated through the MENTUPP Hub, an online platform that presents interactive psychoeducational materials, toolkits, and links to additional resources in an accessible and user-friendly manner (See Figure 2).

Although the MENTUPP intervention was originally planned to be delivered in a hybrid form (i.e., online and face-to-face), all of the materials are delivered online to facilitate participation and engagement during periods of coronavirus disease (COVID-19) restrictions. The material includes online information packages, videos, pre-recorded roleplays, animated scenarios showing case studies, short quizzes, reflection exercises, breathing and mindfulness exercises, and practical stress management exercises. Research shows that having coping strategies may prevent occupational burnout [16]. The Hub is designed to give tailored content to the construction sector, the healthcare sector, and the ICT sector with examples typically experienced within the sectors and presented in videos and animated scenarios by workers from each of the sectors. Within each sector, the Hub provides general material on mental health, wellbeing, and anti-stigmatisation, as well as focused material and tools for employees and for leaders/managers. Participating workplaces are encouraged to employ the materials they engage with in the Hub in their everyday working life.

For the purpose of the pilot study, the MENTUPP Hub will be available in the following languages: Albanian, Dutch, English, German, Hungarian, and Spanish. Materials in the MENTUPP Hub will be delivered to participants via three intervention components that participants will navigate through themselves. Intervention component A focuses on promoting mental wellbeing and targeting non- and pre-clinical mental health aspects including stress, burnout, and depressive symptoms. It involves the presentation of materials that develop the participants’ general understanding and awareness of mental wellbeing, stress, and burnout, as well as a mental wellbeing and stress management toolkit. Leader-specific materials relate to organisational factors associated with wellbeing and teaches communication skills to supervisors on how to address psychosocial aspects of work with employees and develop action plans for modifying the psychosocial work environment. Employee-specific materials relate to stress management and peer support in the workplace with practical guidance on how to build peer support structures supported by video case studies of lived experiences.

Intervention component B is focused on depressive disorders and co-morbid anxiety. It is comprised of psychoeducational materials aimed at gaining a general understanding of what depression and anxiety are, as well as what factors are involved in their onset and maintenance. There are useful tips for users and advice on where to seek further help if necessary. A distinct toolkit for supervisors covers more specific topics including the business impact of these mental illnesses, information on suicide prevention, and guidelines for helping employees in different situations.

Intervention component C aims to target stigma in the workplace and is comprised of psychoeducational materials for all users including lay helpers and sufferers. An overview of the aims, content, and delivery of each component are detailed in Table 1.

### 2.4. Procedure

The research officers in each country will follow the standard operating procedure in the recruitment and implementation of the intervention. Each intervention country will have a local steering committee, comprised of key stakeholders of the sector, experts in workplace mental health promotion, and academia. Potential SMEs will be identified by local steering committees by giving feedback on recruitment (including the identification of potential SMEs) and information materials and facilitating the establishment of links between the local research officer and potential participating organisations. Standard operating procedures will be developed by the research team to guide the recruitment of the companies with specific focus on how to secure commitment from senior management to support and promote the project. The standard operating procedures will also include specifications of minimal commitment expected of companies to participate. To assess if the enterprise is suitable and able to meet the minimum requirements of commitment for participation in the MENTUPP pilot study, the local research officer and a local SME staff member will complete a pre-implementation assessment. The minimum commitments required from SMEs include:

(a) the agreement to develop a project planning group, with the local research officer, local SME representatives, and a local MENTUPP ‘champion’ (a designated employee within the participating SME who will be leading on the promotion, engagement, and implementation of the project),
(b) the understanding that data on individuals will be kept confidential, will not be shared with the SME, and will only be fed back in aggregated form to provide a high-level overview of how participants responded to the survey,
(c) the permission from management for employees to engage with the intervention during paid work hours.

In addition, the pre-implementation assessment will capture the enterprise’s activities in relation to mental health as well as company characteristics such as the number of employees, first language spoken by employees, etc. Once an SME has been recruited, the lead investigator and local research officer will deliver a brief introductory session either face-to-face or online (depending on local COVID-19 restrictions) to employees and employers two weeks prior to intervention implementation. The local research officer will describe the purpose of the study, the scope and nature of the intervention tools, an overview of the questionnaire, and will highlight the opportunity for participants to take part in focus groups after completing the intervention. Employees will be explicitly told that their decision to participate will not impact their position within the company in any way and their details will not be shared back with management of the organisation. All potential participants within an SME will be given an information leaflet with basic information about the study and will be given the opportunity to ask any questions. The information leaflet will provide contact details for the local research officer should they have any questions. Following receipt of this information leaflet, participants will be asked to give consent for participation in the study by completing an online form.

**Table 1:** Overview of the aims, content, and delivery of MENTUPP components.

Module	Aims/Objectives	Material Type(s)	General Material	Sector Specific Versions of the Material	For Employees	For Supervisors
Understanding Mental Wellbeing, Stress and Burnout	To better understand mental wellbeing, stress, and burnout; How to apply 3 approaches for promoting mental wellbeing and preventing mental health problems: proactive, responsive, and reactive; To identify what everyone in the organisation can do to support mental health and wellbeing.	Online written material		YES	YES	YES
Toolkit: Mental Wellbeing and Stress Management	Increase your understanding of mental wellbeing and how it can be strengthened; Increase understanding of stress, how it impacts thoughts and emotions; Introduce practical exercises for managing stress, breathing and mindfulness.	Online written material, reflective exercises, practical exercises		NO	YES	YES
Toolkit: Supervisor Training- Creating Mentally Healthy Workplaces	1. To better understand mental wellbeing, stress, and burnout in the context of the workplace;	Online written material,		YES	NO	YES
	2. To identify and screen for factors in the psychosocial work environment that may be influencing mental wellbeing, stress, and burnout at your workplace;	Reflective exercises,				
	3. To understand how to engage your staff in conversation about psychosocial work environment factors influencing their mental wellbeing, stress, and burnout;	Practical checklists				
	4. To develop action plans to address relevant psychosocial work environment factors influencing mental wellbeing, stress, and burnout in your staff.	Interactive scenarios				
Toolkit: Supporting Each Other at Work	To deepen understanding of peer support and what it entails; Broaden your ability to develop and strengthen it within your workplace.	Online written material, Videos, Practical Exercises Resources		YES	YES	NO
Understanding Depression and anxiety	To better understand depression and anxiety;	Online written material	YES	NO	YES	YES
	2. To learn about the impact of depression and anxiety on work	Videos				
	3. To get to know the different treatment options.	Interactive learning activities				
Toolkit—Supervisor Training—how to address mental illness in the workplace	Understand the business impact of depression and anxiety. Have a conversation with an employee who you think might be depressed or anxious. Support someone who has suicidal thoughts in getting help. Support an employee who has had mental health-related sick leave to successfully return to the workplace.	Online written material, Videos, Practical Exercises	YES	NO	NO	YES
Challenging stigma—a guide for employees and leaders	To better understand stigmatization on the workplace, and how to cope with it.	Online written material	YES	NO	YES	YES
Challenging stigma—a guide for people with mental health issues	To better understand stigmatization, and how to react when being stigmatized. To learn communicational skills to talk about mental health problems.	Online written material	YES	NO	YES	YES
Understanding stigma and discrimination—for all	To better understand stigmatization and possible coping strategies.	Online written material	YES	NO	YES	YES
Test your stigma!	To test the level of stigma.	Online written material	YES	NO	YES	YES

SMEs will meet with the local research officer in their country either face-to-face or online (depending on local COVID-19 restrictions) to discuss what participation entails for the organisation. When an SME agrees to participate in the study, participants from the SME who fulfil the inclusion criteria will be invited to participate in the intervention delivered via the MENTUPP Hub. Participants will be given the opportunity to engage with the materials in the MENTUPP Hub preferably during work hours over a six-month period (in order to view all the content of the Hub, it has been estimated that each user should spend an average of 18–20 min per week). Workplace management will play a role in encouraging engagement with and ‘championing’ the MENTUPP intervention. A pilot planning group will be established within each SME to facilitate the SME in fulfilling their duties in relation to the promotion and engagement of the intervention within the SME. Participants will also have the option of engaging with the intervention in their own free time.

Participants will access the intervention through their personal or work electronic devices (e.g., laptop, computer, smartphone, tablet) at a time of their workday that is allowed by the SME. Participants can also access the intervention from their personal devices at any time of their preference outside of work. Participants will be encouraged to use the skills and knowledge they learn in their working life, in minding their own mental health and in their interactions with their colleagues. Leaders will be encouraged to use the MENTUPP materials in their day-to-day management of the workplace. The pilot planning group within each SME will meet regularly with the research officers to discuss progress in promoting engagement within the SME and to address any issues or barriers that may appear in the process of implementation. The research officers will guide these sessions based on the steps outline in the standard operating procedure. The research officers will feed back to the larger consortium on a weekly basis to present updates and to discuss any issues that arise. Weekly meetings will ensure standardization in addressing any unanticipated issues that may arise during the pilot implementation process. Implementation of the intervention involves flexibility in terms of how the workplace promotes and engages with the MENTUPP Hub. Flexibility is required at this point of the research process given that little is known about what influences managers and what is required for successful implementation of such an intervention [84]. All actions taken as part of the implementation process will be recorded and will contribute to the standard operating procedure of the cRCT at a later point.

### 2.5. Data Collection

Both quantitative and qualitative data will be gathered as part of this pilot study, supporting both pre-post outcome and process evaluations. The data collection and analysis of the pilot study is guided by the RE-AIM framework [29] and will map across the five dimensions of the framework—reach, effectiveness, adoption, implementation, and maintenance. Furthermore, this study will consider the acceptability, appropriateness, and feasibility of the intervention [30].

The process evaluation will examine the process of implementation (whether and how planned activities took place) and the preliminary outcome evaluation will examine whether the expected results and effects of the intervention can be achieved.

#### Process Evaluation

The process evaluation will adopt a combination of the RE-AIM framework [30] and the evaluation framework outlined by Proctor and colleagues [31]. Data will be gathered from multiple sources: self-developed surveys, validated scales, a monitoring instrument, online log data of users, and focus groups with leaders, employees, and local research officers responsible for implementing the MENTUPP intervention in the intervention countries. The Organizational Culture Assessment Instrument (OCAI) [32] will be administered at baseline only from a selection of managers and will be used to understand the culture of, and the presence of psychosocial risk factors and stressors in the SMEs. Information at organisational level will be collected from a designated employee in a supervisory or management position from each SME using a monitoring instrument developed by the MENTUPP consortium. The instrument collects quantitative and qualitative information of the SME at the time of recruitment (general information on the SME and staff, experience with mental health problems in the SME, how the SME deals with employee mental health issues, the impact of COVID-19 on the SME) and information on the adoption and implementation of the MENTUPP intervention throughout the pilot study (relevant events or changes in the SME and the country, relevant information with respect to the planned activities, etc.).

Three surveys including internationally validated measures tailored to the specific evaluation needs of the MENTUPP pilot study will be used to collect information at the individual level at baseline and follow-up. The pre-intervention survey will be administered to all employees and gather information relating to the following themes: sociodemographic and work-related information; ways of approaching mental health difficulties in the organisation; experience with mental health difficulties; expectations of the MENTUPP intervention; acceptability, appropriateness, and feasibility of MENTUPP at baseline and follow-up; and the impact of COVID-19 on mental health and the workplace. A post-intervention survey for employees will ask participants for specific feedback on their views and experiences of participating in MENTUPP and on their views of the MENTUPP Hub on completion of the intervention period. A post-intervention survey for leaders will assess the extent to which activities recommended by MENTUPP are initiated in the SME (e.g., better work planning, more peer support, regular meetings, etc.).

Log data on user activity tracked via the MENTUPP Hub will be collected to compile information on how frequently the MENTUPP Hub was used and which parts of the platform were most frequently accessed.

Internationally validated questionnaires (listed below) will be administered during facilitated sessions at baseline before access to the MENTUPP Hub is given, and again at the end of the 6-month pilot intervention period. The following self-report, standardised measures of wellbeing and mental health will be included:

○ Mental wellbeing and quality of life: The World Health Organisation—Five Wellbeing Index (WHO-5) [85]
○ Depression and anxiety: Patient Health Questionnaire Anxiety and Depression Scale (PHQ-ADS) [86]
○ Depression stigma: Depression Stigma Scale (DSS) [87]
○ Presenteeism in the workplace: Stanford Presenteeism Scale (SPS-6) [88]
○ Productivity: Work Productivity and Activity Impairment—General Health V2.0 (WPAI-GH 2.0) [89]
○ Burnout: Oldenburg Burnout Inventory (OLBI) [38,90]
○ Help-seeking behaviour: Attitudes Towards Seeking Professional Psychological Help-Short Form (ATSPPH) [91]
○ Presence (level) of psychosocial risk factors and stressors: Selected items and scales from the Copenhagen Psychosocial Questionnaire (COPSOQ) [92]

Following the intervention period, focus groups will be conducted to gain more in-depth qualitative information on the quality and intensity of the pilot implementation of the intervention in each of the intervention countries. Information on the perspectives and experiences of employees and employers who engaged with the intervention, as well as the barriers and facilitators to implementation of the intervention will be obtained. Separate focus groups will be conducted with employees and with supervisors/managers in the participating SMEs. These focus groups will provide in-depth information with respect to the strengths and the weaknesses of the content of the MENTUPP Hub including the acceptability, comprehensibility, feasibility, appropriateness, relatability and uptake of the content, and barriers and facilitators of the implementation of the MENTUPP intervention tools. There will be also a focus group with the local research officers of each country, to discuss the experiences with the recruitment of SMEs, barriers, facilitators, and experiences of, implementation, and areas of improvement for the implementation and evaluation of the MENTUPP intervention. In every country, at least one focus group with 3–5 leaders across organisations (i.e., managers, supervisors, health and safety professionals) and one focus group with 8–10 employees will be organised based on previous research with focus groups [93]. These focus groups will discuss communication of the MENTUPP intervention within the SME, experiences of using the MENTUPP Hub, areas for improvement to either the intervention, evaluation, or both, and the extent to which activities recommended were initiated in the workplace.

### 2.6. Data Management

The survey data will be collected via Qualtrics (Provo, UT), which is a General Data Protection Regulation (GDPR) compliant online platform. Participants will create their own unique identifier that will allow researchers to link their evaluation data to their activity on the MENTUPP Hub. Any personal information collected (e.g., email address/phone number) required in accessing the Hub will be stored separately from evaluation data. The information that participants provide will be aggregated for analysis by SME/sector depending on the number of cases and an individual’s data will not be fed back to SME management. Qualitative data will be collected by local research officers.

### 2.7. Data Analysis

#### Process and Preliminary Outcome Evaluation

The self-reported surveys (including validated questionnaires) and monitoring instruments collected pre- and post-intervention will be analysed using descriptive statistics and non-parametric and parametric tests. Patterns of completeness in the survey will inform the development of the self-reported surveys (including validated questionnaires) to be used in the cRCT. The log data from the MENTUPP Hub will also be analysed to establish patterns of engagement by calculating the number of participants visiting the Hub more than once, the total number of visits to the Hub overall and on an individual level, and the total time spent accessing and engaging with the content.

Qualitative data of the focus groups will be audio recorded, transcribed, and analysed using thematic analysis [94] to explore patterns in qualitative data across participants and across sectors. Themes and subthemes will be determined as the overarching categories of common data across multiple participants.

Quantitative and qualitative data will be interpreted in relation to each sector as well as each intervention country to understand the implementation of the intervention within each context. This information will be used to optimise the intervention for the cRCT.

### 2.8. Ethical Considerations

The present study has been approved by each of the local research officer’s institutional ethics committees and is registered with ISRCTN clinical trial registry (ISRCTN14582090).

#### 2.8.1. Duty of Care

Given the potential stigma and risk associated with mental health issues, particular care will be taken when members of the project teams engage with employees and employers with mental health issues after informed consent has been received. Although the target groups in the project are comprised of individuals who may be vulnerable, previous European Alliance Against Depression projects have found the interventions being offered to have beneficial effects on mental health without any adverse effects. Therefore, the benefits of participating are expected to outweigh any potential risks. The PHQ-ADS, administered at baseline and follow up, includes an item which addresses suicidal ideation. Participants will be presented with a message at the end of the survey encouraging them to contact their general practitioner (GP), local mental health services or quality-assured support service, or the local research officer if they reported that they have experienced suicidal ideation. Participants in the study will be provided with contact details for the local research officers should they require support. Local research officers and other personnel involved in direct contact with employees and employers in the pilot study will receive special training in relation to general mental health difficulties, and more specifically, depression, anxiety, and suicidal behaviour. They will be provided with supervision in their work setting by the lead investigators in each of the intervention countries on an on-going basis.

#### 2.8.2. Data Protection

Personal data, including participant name, will be collected to ensure a record is kept of all participants who give their informed consent to participation in the research study. However, personal data, including participant name, will be stored separately from the evaluation data they provide, will not be shared with third parties, or appear in any reports. Each participant will have a unique identification code which is the combination of responses to four personal questions which is generated to ensure data will be kept as confidential as possible [41]. It will be used (1) to link the user activity data with the evaluation data; (2) to link participant data from different time points; and (3) to ensure accuracy in the dataset by allowing the participant to access, correct, or withdraw any data that they submit to the study. Informed consent will also be recorded in compliance with GDPR. Data will be controlled and managed by MENTUPP consortium members based at KU Leuven.

## 3. Discussion

The MENTUPP intervention employs a multilevel approach that focusses on educating supervisors and employees about mental health at work, gives tools to supervisors that can be used to promote mental wellbeing at work and reduce risk factors in the psychosocial work environment, as well as individual oriented tools for coping with stress and supporting peers. Although multilevel approaches are often recommended, high quality studies testing multilevel approaches at the workplace are largely missing [36,70,95,96,97].

The MENTUPP intervention has been developed based on theoretical frameworks [22,23] by leading researchers in occupational mental health from across the EU and Australia. The intervention has involved the co-operation and consultation of key stakeholders in occupational health in the three occupational sectors to ensure that the tools are relevant and specific to their needs. The approach also involves SME management from the very beginning of the implementation of the MENTUPP intervention to encourage support for and uptake of organisational level approaches to improve mental health of employees in SMEs.

The results of this pilot study will provide a comprehensive overview of the implementation of such an intervention in a variety of contexts, languages, and cultures. The overall MENTUPP project will not only provide evidence-informed, tailored tools for employees and managers/owners of SMEs but will evaluate how useful the tools are and better understand the barriers for using these tools in real-life settings across the healthcare, construction, and ICT sectors. Furthermore, given that construction is a male-dominated industry and healthcare is generally female-dominated, this provides us with an added opportunity to understand the implementation of such an intervention with consideration of potential gender differences.

Adverse effects of participating in the pilot study include the effect of potential mental health related stigma and the risks associated with mental health issues [98]. Participants might be hesitant to participate in the pilot study as participation might be perceived to harm their employment. This risk will be mitigated by SME engagement with the online delivery of the MENTUPP Hub that can also be accessed in participants’ own time, and the strict confidentiality with which participant data will be treated with.

### Strengths and Limitations

There are several strengths and limitations associated with this pilot study. The MENTUPP intervention is tailor-designed through systematic application of theory and evidence for workers in three vulnerable occupation sectors. However, these specific job stressors may act as a barrier to engagement with the MENTUPP intervention. As mentioned previously, COVID-19 may also increase burden on SMEs, and this may implicate significant time and resource constraints on SMEs and employees within SMEs which may prevent them from deciding to participate in the programme or may hinder them from engaging with the programme as needed [99]. COVID-19 and associated restrictions may also be a barrier to the recruitment and implementation of the intervention given that face-to-face interaction may not be possible. Therefore, the outcomes of this pilot study may provide a distorted view of how the intervention may be implemented given the decentralised/interrupted workforce. To help overcome the impact of job stressors and COVID-19 on engagement, the MENTUPP intervention has been designed with flexibility in its implementation (e.g., self-directed online materials) to engage SMEs and individual participants to its maximum potential.

Furthermore, this study employs both quantitative and qualitative data collection methods to obtain a comprehensive understanding of the reach, effectiveness, accept-ability, appropriateness, feasibility, implementation, and maintenance of the MENTUPP intervention. This evidence-based online programme is designed to target all levels of an organisation. However, it is possible that self-selection by the SMEs and further, the employees may influence the uptake and the outcomes and may understate the overall effectiveness of the intervention in the workplace setting. Self-report measures are used to capture potential changes and these may be inaccurate or incomplete. We may also be limited in our ability to assess the psychometric properties of the measures given the smaller samples for each of the languages. Furthermore, the flexible approach to implementing the intervention at this early stage of the intervention development may result in variability in how SMEs are invited and recruited (reach), and in how the intervention is implemented in the workplace (implementation). This step is necessary in learning about the various approaches to maximizing reach and to successfully implementing the intervention in the workplace, in consultation with the SMEs themselves, ahead of the cRCT. Nevertheless, considering that more research on mental health promotion in SMEs is needed, we believe that this pilot study will provide us with crucial knowledge about the needs and possibilities for improving mental health in smaller workplaces across Europe and Australia.

## 4. Conclusions

The findings from this pilot study will inform the implementation and evaluation of a cRCT of the MENTUPP intervention in three occupational sectors, in nine intervention countries across Europe and Australia. Due to the high rates of mental health problems and suicide in the targeted sectors of construction, health, and ICT, the MENTUPP intervention programme is both timely and relevant, and in particular considering the COVID-19 related impacts on mental health in the workplace.

## Data Availability

No new data were created or analysed in this article. Data sharing is not applicable to this article.
